# Functional characterization of open chromatin in bidirectional promoters of rice

**DOI:** 10.1038/srep32088

**Published:** 2016-08-25

**Authors:** Yuan Fang, Ximeng Wang, Lei Wang, Xiucai Pan, Jin Xiao, Xiu-e Wang, Yufeng Wu, Wenli Zhang

**Affiliations:** 1 State Key Laboratory for Crop Genetics and Germplasm Enhancement, JiangSu Collaborative Innovation Center for Modern Crop Production (JCIC-MCP), Nanjing Agriculture University, Nanjing, Jiangsu 210095, China

## Abstract

Bidirectional gene pairs tend to be highly coregulated and function in similar biological processes in eukaryotic genomes. Structural features and functional consequences of bidirectional promoters (BDPs) have received considerable attention among diverse species. However, the underlying mechanisms responsible for the bidirectional transcription and coexpression of BDPs remain poorly understood in plants. In this study, we integrated DNase-seq, RNA-seq, ChIP-seq and MNase-seq data and investigated the effect of physical DNase I hypersensitive site (DHS) positions on the transcription of rice BDPs. We found that the physical position of a DHS relative to the TSS of bidirectional gene pairs can affect the expression of the corresponding genes: the closer a DHS is to the TSS, the higher is the expression level of the genes. Most importantly, we observed that the distribution of DHSs plays a significant role in the regulation of transcription and the coexpression of gene pairs, which are possibly mediated by orchestrating the positioning of histone marks and canonical nucleosomes around BDPs. Our results demonstrate that the combined actions of chromatin structures with DHSs, which contain functional cis-elements for interaction with transcriptional machinery, may play an important role in the regulation of the bidirectional transcription or coexpression in rice BDPs. Our findings may help to enhance the understanding of DHSs in the regulation of bidirectional gene pairs.

Bidirectional gene pairs have biological significance in both mammalian and plant systems; they function in basic biological processes in humans, including DNA repair, cell cycle, housekeeping, metabolic pathways and human diseases^1^,^2^,^3^,^4^,^5^,^6^,^7^,^8^,^9^,^10^,^11^,^12^. Similarly, plant BDPs function in the regulation of important agricultural traits^13^,^14^,^15^,^16^,^17^,^18^. The transcription of bidirectional protein-coding gene pairs arranged with in a head-to-head orientation is controlled by bidirectional promoters (BDPs), which have been intensively investigated in eukaryotic genomes, ranging from yeast^19^,^20^, *Drosophila*^21^, and humans^5^,^22^ to plants^23^,^24^. Compared to unidirectional promoters (UDPs), more enriched RNA PolII binding; acetylation at H3, H3K9 and H3K27; and methylation at H3K4me2/3 were observed in human BDPs^25^,^26^; in contrast,H4 acetylation was less enriched^27^, indicating that BDPs may possess unique characteristic chromatin features that are responsible for the regulation of human BDPs.

With the release of whole-genome sequencing and transcriptomic data in plants, plant BDPs have already received considerable attention. So far, BDPs have been investigated in *Arabidopsis*^5^,^28^,^29^,^30^, rice^23^, maize^24^ and *Populus*^23^. The sequence features are well conserved between mammalian and plant genomes^23^,^28^,^31^,^32^. However, it remains unclear the epigenetic mechanisms for the bidirectional transcription and coexpression of gene pairs in plants.

In this study, we integrated DNase-seq, RNA-seq, ChIP-seq and nucleosome positioning data and investigated the effect of DNase I hypersensitive sites (DHSs) on the transcription of rice BDPs. We found that the physical position of a DHS relative to the TSS of bidirectional gene pairs can affect the expression of the corresponding genes: the closer a DHS is to the TSS, the higher is the expression level of the genes. Most importantly, we observed that the DHS distribution plays a significant role in the regulation of transcription and the coexpression of gene pairs, possibly mediated by orchestrating the positioning of histone marks and canonical nucleosomes around BDPs.

## Results

### Distribution of DNaseI hypersensitive sites in rice BDPs

In this study, we identified a total of 290, 294 and 627 gene pairs corresponding to the BDP sizes of 0–250 bp (BDPs I), 250–500 bp (BDPII) and 500–1000 bp (BDPsIII), respectively, using the updated version 7.0 released from the Institute for Genomic Research (TIGR) rice (subsp. *Japonica*), containing a total of 55,801 annotated genes.

DNaseI hypersensitive sites (DHSs) are considered as markers to identify cis-regulatory elements (CREs), such as promoters and enhancers^33^,^34^,^35^,^36^. To profile DHSs within rice BDPs, we plotted normalized DNase-seq reads across BDPs and performed DH peak calling. According to the distribution of DHSs, we divided BDPs into four categories: one single-DHS located almost in the middle of a BDP (**one mid-DHS**); one single-DHS located closer to one gene than the other (**one amesial DHS**); two DHSs located in a BDP (**bi-DHSs**) and no detectable DHS (**no DHS**) (Supplementary Fig. S1). The percentage of one mid-DHS dramatically decreased from BDPs I to BDPs III (78.27% in BDPs I vs. 4.31% in BDPs III) (Table 1). In contrast, the percentage of the other three DHS categories increased from BDPs I to BDPs III, even though the difference between one amesial DHS and no DHS in BDPsII and III was subtle (Table 1).

To verify whether one DHS truly represents a functional BDP responsible for the transcription of bidirectional gene pairs, we performed rice leaf protoplast-based transient transformation using GFP as a reporter gene. We observed the green GFP signal from the inserted vector, regardless of its conformation (forward or reverse) (Supplementary Fig. S2).We randomly selected five BDPs containing one DHS, four of which were experimentally verified as BDPs. When combined with 7 experimentally verified rice BDPs containing one DHS^31^, 10 of the 12 (83%) were BDPs and only two were UDPs (Supplementary Table S1), possibly due to the existence of an insulator or repressor blocking the promoter activity in the other direction or they actually function as UDPs.

Taken together, DHS profiling combined with transient validation demonstrates that BDPs consist of either one promoter functioning in bidirectional transcription or two individual unidirectional promoters physically located close to each other but functionally control the transcription of the corresponding downstream gene.

### Effect of DHSs on the expression of bidirectional gene pairs

**Figure 1:**
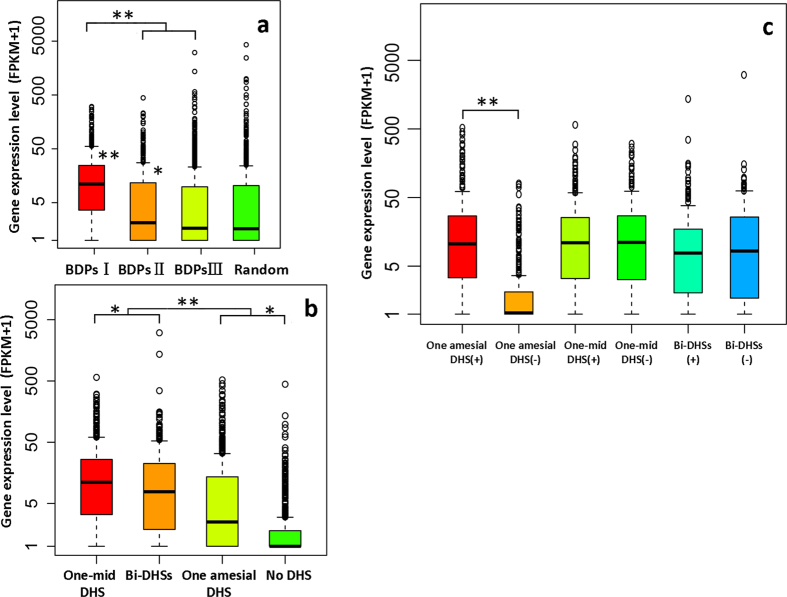
Comparison of the expression level of gene pairs. FPKM values were used to indicate the expression level of each gene pair. A significance test was performed using a two-sample K-S test to indicate whether the expression level between two samples differed significantly. The X-axes show both BDP genes and UDPs control in (**a)**, BDPs with a different physical position of DHS relative to the TSS of BDP genes in (**b)**, and genes with a higher FPKM (+) and a lower FPKM (−) associated with BDPs with a different DHS distribution in **(c)**; the Y-axes are log scale with FPKM +1 values. (**a**) Comparison of gene pairs in each BDP. **p < 2.2e-16 and *p < 0.05. (**b**) Comparison of gene pairs associated with BDPs containing different DHS distributions. **p < 1e-11 and *p < 0.05. (**c**) Comparison between genes with a higher FPKM (+) and lower FPKM (−) associated with BDPs with different DHS distributions. The positive sign “**+**” represents a higher FPKM; the negative sign “−” represents a lower FPKM. The expression level of genes located proximal to the DHS peak is significantly higher than that of genes e located distal to the DHS peak (**p < 2.2e-16).

**Figure 2:**
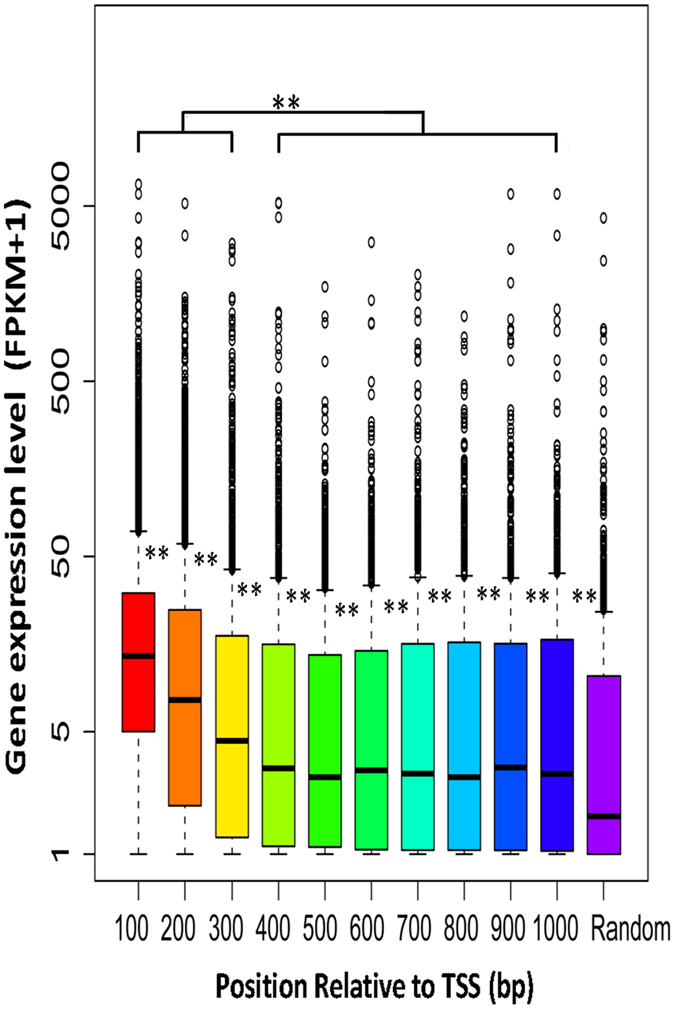
The relationship between the distance of DHS to TSS and gene expression. All of the genes with one DHS peak located within 1 kb of the TSS were selected to compare their expression levels. The distance between the DHS peak and TSS was calculated from the midpoint of the DHS peak to the TSS. The X-axes show the distance from the DHS peak to the TSS at every 100 bp intervals; the Y-axes are log scale with FPKM +1 values. A statistical analysis was performed using two-sample K-S test, where **p < 0.001.

DHSs usually harbor functional CREs, which are responsible for the regulation of gene expression across eukaryotic genomes. From the relationship between BDPs and the expression level of the corresponding gene pairs (Fig. 1a), we found that the expression level of gene pairs in BDPs I was significantly higher than that of gene pairs from the other two BDPs II (*p*-value < 2.2e-16 for BDPs II and *p*-value < 2.2e-16 for BDPs III, K-S test) and randomly selected unidirectional genes (*p*-value < 2.2e-16, K-S test). Additionally, the expression level of gene pairs in BDPs II was significantly higher than that of gene pairs from randomly selected unidirectional genes (*p*-value = 0.04971, K-S test), but there was no significant difference between BDPs III and UDPs, or between BDPsII and BDPsIII (Fig. 1a). This result indicates that the expression of bidirectional gene pairs decreases with the increased intergenic distance among three BDPs. We then investigated the effect of the physical position of DHSs relative to the TSS of bidirectional gene pairs on the expression of the corresponding genes. Clearly decreasing expression was observed from gene pairs with one mid-DHS having the highest expression (mean of FPKM value is 10.11) to gene pairs with no DHS having the lowest expression (mean of FPKM value is 0.00) (Fig. 1b). Furthermore, no significant difference in expression level was observed in gene pairs containing either one mid-DHS or bi-DHSs; in BDPs containing one amesial DHS, however, the expression level of the gene located proximal to the DHS was significantly higher than that of the counterpart located distal to the DHS (*p*-value < 2.2e-16, K-S test) (Fig. 1c). It seems that gene expression is highly associated with the physical position of DHSs relative to the TSS of the corresponding gene. To verify whether this phenomenon also exists in unidirectional genes genome-wide, we first extracted all of the expressed unidirectional genes; we then grouped all of these genes according to the physical position of the DHS relative to the TSS of the corresponding genes separated by every 100 bp and analyzed the expression level of genes within each group based on the FPKM value of each gene. We randomly selected 1000 genes regardless of the physical position of the DHS relative to TSS of the genes and analyzed the expression levels as a control (Fig. 2). We finally investigated the relationship between the physical position of a DHS relative to the TSS of genes and the expression level of the corresponding genes. In general, we found that unidirectional genes with one DHS located 1 kb upstream of TSS displayed significantly higher expression than did randomly selected genes (Fig. 2). Compared to randomly selected genes, the K-S test showed *p*-value < 2.2e-16 and *p*-value = 4.439e-05 for genes with DHSs located 100 bp and 1000 bp away from TSS, respectively. Strikingly, genes with a DHS located less than 300 bp from TSS showed significantly higher expression than did others; the highest expression level was found in genes with a DHS located 100 bp from TSS (mean of FPKM: 12.52) (Fig. 2). Compared to genes with a DHS located 1000 bp from TSS, the K-S test showed *p*-value < 2.2e-16 and *p*-value = 1.981e-06 for genes with DHSs located 100 bp and 300 bp from TSS, respectively. Thus, genes with a DHS located less than 200 bp from TSS show a higher expression level than did others (both UDP and BDPs genes). These results demonstrate that the physical position of DHSs relative to the TSS of genes can affect the expression of the corresponding genes: the closer a DHS is to the TSS, the higher is the expression level of the genes. This result indicates that different regulation modes may exist for the regulation of gene expression associated with proximal and distal promoters within the genome.

### Effect of DHSs on the coexpression of bidirectional gene pairs

**Figure 3:**
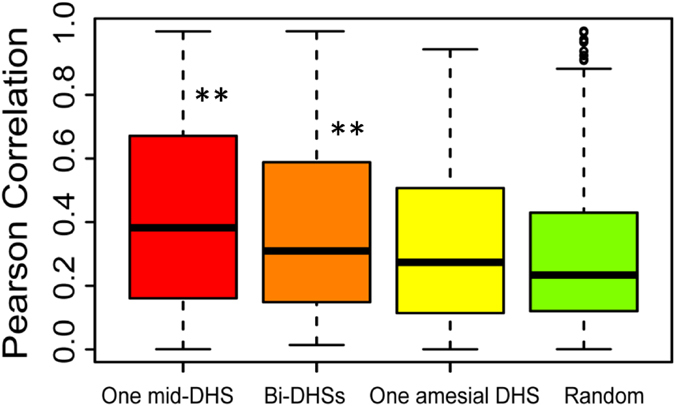
Effect of the DHS profile on the coexpression of bidirectional gene pairs. The presence of DHS within BDPs was classified into three categories according to its physical distance relative to the TSS of the genes: one mid-DHS, bi-DHSs, and one amesial-DHS. Then, 1000 randomly selected UDP genes were used as controls. The Pearson correlation coefficient was used to indicate the coexpression of bidirectional genes, as calculated from all of the gene pairs using the absolute expression value. Statistical analysis was provided by a two-sample K-S test, where **p < 0.001.

To analyze the coexpression of bidirectional gene pairs, we extracted 11 gene expression datasets from the Rice Genome Annotation Project (Supplementary Table S2) (http://rice.plantbiology.msu.edu/expression.shtml) to calculate the Pearson correlation coefficients. We then categorized bidirectional gene pairs in terms of their intergenic interval as 100 bp and analyzed the percentage of co-expressed gene pairs separated by every 100 bp interval (Supplementary Table S3), we observed that the percentage of co-expressed gene pairs was higher in BDPs with intergenic distances of less than 300 bp, which surprisingly contain the highest percentage of one mid-DHS (Supplementary Table S4). This result agrees with the strongest coexpression levels found in gene pairs separated by 200 bp (Supplementary Fig. S3). We suspected that the physical position of a DHS relative to the TSS of bidirectional genes may affect the expression mode of bidirectional gene pairs. To test this hypothesis, we conducted a correlation analysis between DHS distribution and the coexpression of bidirectional gene pairs. Compared to randomly selected unidirectional genes, we indeed observed that the coexpression of bidirectional gene pairs was highly correlated with BDPs containing either one mid-DHS (*p*-value = 4.44e-11, K-S test) or bi-DHSs (*p*-value = 9.85e-05, K-S test), but no significant correlation was observed in BDPs containing one amesial DHS (Fig. 3). When comparing one mid-DHS, bi-DHSs and one amesial DHS, a significant correlation was observed in BDPs containing one mid-DHS and one amesial DHS (*p*-value = 4.66e-04, K-S test) (Fig. 3). These analyses indicate that the physical position of a DHS relative to the TSS of bidirectional genes might affect the coexpression of bidirectional gene pairs.

To investigate the functional consequences of BDPs containing different physical positions of DHSs, we further performed a GO analysis (data not shown) and found that gene pairs containing different locations of DHSs function in different biological functions. For example, bidirectional gene pairs with one amesial DHS, bi-DHSs and one mid-DHS are associated with GO terms with functions in cytoplasm; gene expression, intracellular part and cytoplasm; as well as cell part and intracellular part, respectively. This pattern is especially true in gene pairs without detectable DHSs that are mainly responsible for apoptosis and for the transport and localization of lipids, indicating that gene pairs with DHSs in the same position have similar associated GO terms. Thus, all of the above analyses demonstrate that the physical position of a DHS relative to the TSS of bidirectional genes plays a significant role in the regulation of gene pairs’ transcription and coexpression.

### Effect of DHSs on nucleosome positioning

**Figure 4:**
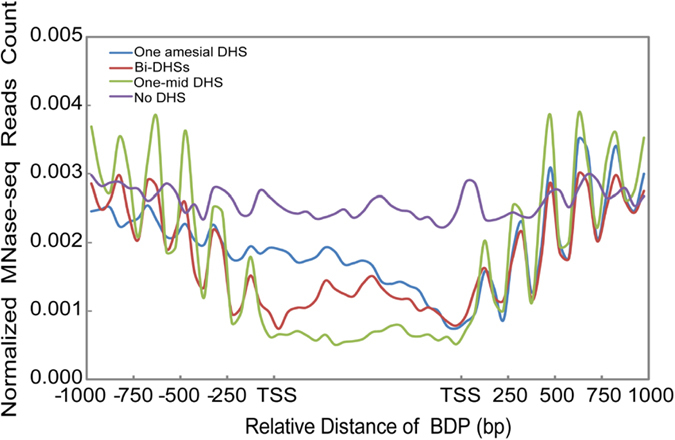
Profile of nucleosome positioning around BDPs containing the different physical distances of DHS relative to the TSS of genes. Bidirectional gene pairs with higher and lower FPKM values were aligned on the right and left sides of BDPs, respectively. The normalized MNase-seq reads count representing the nucleosome positioning was calculated by the numbers of reads per base pair in a genomic region per million reads. The X-axes show the relative distance of BDPs (bp); The Y-axes show normalized MNase-seq reads counts (read number per base pair in a genomic region per million reads) within ±1 kb of the TSS. Paired-end MNase-seq reads were used to profile the nucleosome positioning after normalization. The bottom diagram indicates the direction of different expression levels from each gene pair: the highly expressed genes (higher FPKM values) are located on the right side, and the lowly expressed genes (lower FPKM values) are located on the left side.

**Figure 5:**
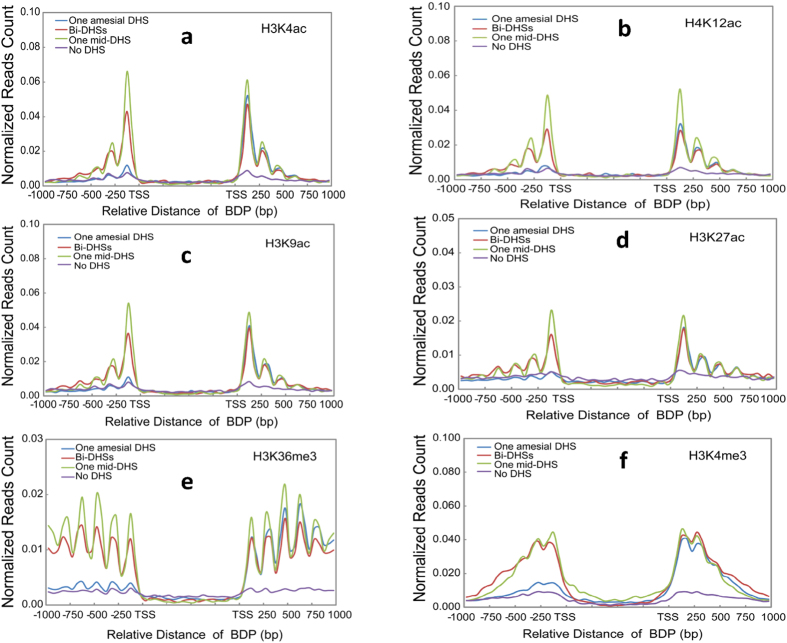
Effect of DHS on the positioning of active histone marks. Effect of the physical position of a DHS relative to the TSS of genes on the positioning of parts of active marks: (**a**) H3K4ac, (**b**) H4K12ac, (**c**) H3K9ac, (**d**) H3K27ac, (**e**) H3K36me3 and (**f**) H3K4me3. The X-axes in (**a**–**f**) show the relative distance of BDPs (bp); the Y-axes in (**a**–**f**) show normalized ChIP-seq reads counts (read number per base pair in a genomic region per million reads) within ±1 kb of the TSS. In general, ChIP-seq reads counts of histone marks H3K27ac (**5d**) and H3K36me3 (**5e**) are relatively lower than others. We used different y-axis scales in both plots to better visualize the profile of both marks distributed among bidirectional promoters containing different DHSs.

In eukaryotes, local or global changes in chromatin structure mediated by nucleosome remodeling or histone modifications result in the presence or absence of open chromatins, which are hypersensitive to DNaseI cleavage (DHSs). Chromatin changes directly or indirectly affect a series of biological processes, including transcription, replication and repair^37^. The effect of chromatin remodeling on the coexpression of gene pairs has been observed in yeast^38^. To determine whether there exists an interplay between DHSs and nucleosome positioning in BDPs, we examined the effect of the physical position of DHSs on the nucleosome positioning around BDPs. Well-oscillated nucleosomes symmetrically flanked BDPs with one mid-DHS and bi-DHSs and further extended to the corresponding gene body (Fig. 4). Furthermore, the highest amplitude of nucleosome was found in BDPs with one mid-DHS. Interestingly, nucleosomes around BDPs with one amesial DHS were more positioned to the side proximal to the DHS than to that distal to the DHS, displaying DHS-mediated nucleosome positioning (Fig. 4). Similarly, a significant effect of DHS on the positioning of modified nucleosomes was observed in active histone marks (acetylation at H3K4/K9/K27 and H4K12 and methylation at H3K4/K36) (Fig. 5a–f, Supplementary Fig. S5d),which favor gene transcription, but there was almost no effect on the positioning of the repressive mark-methylation atH3K27/K9 (Supplementary Fig. S5a–c), which disfavors gene transcription. Thus, combined with the findings above that the coexpression of bidirectional gene pairs was highly associated with BDPs containing either one mid-DHS (*p*-value = 4.44e-11, K-S test) or bi-DHSs (*p*-value = 9.85e-05, K-S test), these results indicate that DHSs play significant roles in the regulation of transcription and the coexpression of gene pairs, possibly mediated by orchestrating the positioning of histone marks and canonical nucleosomes around BDPs.

## Discussion

DHS sensitivity is directly correlated with the expression level of unidirectional genes in eukaryotic genomes^33^,^36^. However, the relationship between DHSs and the expression of bidirectional gene pairs is still unclear. In this study, rice gene pairs with BDPs containing either one mid-DHS or bi-DHSs display a significant coexpression level compared to that of randomly selected UDPs and one amesial DHS-BDPs, indicating that the physical position of DHSs within rice BDPs affects the transcription mode of bidirectional gene pairs. The symmetric position of DHSs within a promoter region may be a key player in the coregulation of bidirectional gene pairs. DHSs in the promoter region usually harbor cis-regulatory elements for the binding of RNA polymerase II and other transcription machinery, and are thus involved in the regulation of the gene transcription^39^. We speculate that the symmetric distribution of DHSs (either one-mid DHS or bi-DHSs) within rice BDPs play two possible roles in the coregulation of gene pairs. One role is that the presence of DHSs represents the open chromatin region, which may simultaneously facilitate the expression of gene pairs. The other role is that gene pairs may be controlled by the same transcriptional machinery with bi-directionally equal efficiency due to sharing the same regulatory elements. Similar chromatin structure-based mechanisms responsible for the coregulation of gene pairs have been reported in the mammalian genome^22^,^40^. On the other hand, bidirectional promoters are identified based on expressed adjacent gene pairs, which are organized in a divergent fashion and physically separated by less than a 1 kb interval, but *in vitro* transient transformation results showed that about 17% of them unexpectedly function as UDP inducing unidirectional expression of the reporter gene. We suspected the possible reasons as below: first the expression of gene pairs is possibly regulated by different distal cis-elements, thus the absence of related cis elements in the tested DNA fragment can affect the expression of the corresponding gene resulting in unidirectional expression. Deletion based verification demonstrates that the presence of cis-elements, like enhancers, repressors or insulators, is essential for the function of rice BDPs^31^. Secondly, we can not exclude the possibility that some of BDPs are misclassified and function as real UDPs. Thus, it is necessary to validate any of predicted BDPs before further application or analysis of them.

The involvement of nucleosome positioning on gene expression or the evolution of gene regulation has been intensively studied in eukaryotes^41^,^42^,^43^,^44^,^45^,^46^,^47^,^48^. However, little is known about the effect of chromatin organization on the regulation of coexpressed gene pairs in plants. At the chromatin level, well-oscillated nucleosomes are symmetrically distributed around rice BDPs, which contain either one mid-DHS or bi-DHSs; in particular, −1 and +1 nucleosomes are highly phased in BDPs with one mid-DHS or bi-DHSs. In contrast, in BDPs with one amesial DHS, a higher occupancy of well- positioned nucleosomes was only present in the gene with the TSS closer to the DHS than to the other side. A similar DHS-directed positioning occurs in active histone marks. Interestingly, the expression level of rice gene pairs is closely related to the positioning and occupancy of nucleosomes around rice BDPs, which contain either one mid-DHS, bi-DHSs or one amesial DHS. Similarly, the occupancy and positioning of active marks instead of repressive marks display a high association with rice gene expression genome-wide (Supplementary Fig. S6). These results indicate that the presence of well-positioned nucleosomes around rice BDPs may facilitate the expression of the corresponding (co)expressed genes, possibly mediated by the regulation of transcription initiation or elongation. Histone modifications affect the binding of transcription factors in DHSs^49^, and the chromatin structure plays a key role in regulating the expression of clustered genes in mammalians^40^,^50^. A possible mechanism for the effect of histone modification on gene expression has been proposed in mammalian and yeast genomes. It has been proposed that gene transcription can be regulated either at the initiation step or during the elongation process^51^,^52^. Both steps can be influenced by histone marks residing in the promoter and gene body regions^53^. The promoter-related active marks H3K4me3 and H3K9/K14 ac and the gene-body-related active mark H3K36me3 are associated with transcription initiation and elongation in the mammalian and yeast genomes^54^,^55^,^56^, respectively, possibly by affecting Pol II movement along chromatin directly or indirectly^57^,^58^,^59^. Thus, active marks that are enriched either at the transcription initiation step (H3K4me3 and acetylation at H3K4/K9/K27 and H4K12) or at the elongation step (H3K36me3) may coordinate the presence of stalled or elongating RNA polymerase II.

Rice BDPs only containing a symmetric presence of DHS are flanked by well-positioned canonical and active mark (H3K4me3, H3K36me3, acetylation at H3K27/4/9 and H4K12ac)-related nucleosomes, indicating that the physical position of DHSs plays a significant role in the positioning of canonical nucleosomes and active marks around rice BDPs, thereby facilitating the expression of the corresponding (co)expressed genes. Orchestration between DHSs and nucleosome positioning has been previously characterized in rice^60^, and DHSs flanked by well- positioned nucleosomes have been observed in rice, *Arabidopsis* and human genomes^48^,^60^. Because the positioning of nucleosomes around rice BDPs is closely related to the physical distance between the DHSs and TSS of the genes, we speculate that the binding of RNA polymerase II and other transcription machinery to DHSs may be a key determinant for the nucleosome positioning of canonical or active marks in rice BDPs. Transcription factors, chromatin remodelers and RNA polymerase play key roles in the positioning of nucleosomes in yeast and humans^48^,^61^,^62^. The binding of basal transcription factor-like pre-initiation complexes to the core promoter may help to initiate and maintain a well-positioned +1 nucleosome in yeast^63^,^64^. In human CD4 + T cells, either stalled Pol II or elongating Pol II is associated with the presence of a +1 nucleosome located within a certain distance downstream of TSS^42^. Elongating Pol II machinery can establish a nucleosome array in coding regions in yeast^65^. Thus, the effect of DHSs on the positioning of nucleosomes may be mediated by the recruitment of transcription machinery, including transcription factor, chromatin remodeler and Pol II.

Combined with coexpression associated with one mid-DHS and bi-DHSs, we conclude that the symmetric presence of a well-positioned canonical nucleosome, as well as active histone mark may create chromatin structures favoring the coexpression of gene pairs. On the other hand, we first found that the closer a DHS is to the TSS, the higher is the expression level of the genes, which was observed in 83.7% of gene pairs associated with BDPs containing one amesial DHS (Supplementary Fig. S5e) and unidirectional genes (Fig. 2). Taken together, our results demonstrate that the physical position of DHSs plays a significant role in the expression and coregulation of gene pairs, which may be achieved by orchestrating the positioning of canonical nucleosomes and active histone marks around BDPs.

## Materials and Methods

### Collection of rice seedlings

Rice cultivar “Nipponbare” seeds were germinated and grown in a greenhouse. Two-week-old rice seedlings were collected for the ChIP-seq experiments below.

### Identification of bidirectional promoters in rice

Rice (*Oryza sativa*, subsp *japonica*) genomic sequence and annotation datasets were extracted from the Rice Genome Annotation Database at TIGR (http://www.tigr.org/tdb/e2k1/osa1). Bidirectional gene pairs with head-to-head orientation were identified. The intergenic regions between the TSS of each gene pair were designated as bidirectional promoters (BDPs). BDPs were classified into three categories: 0–250 bp (BDPs I), 250–500 bp (BDPs II) and 500–1000 bp (BDPs III). All of the gene pairs that were annotated as protein-coding genes were included for the downstream analysis. For comparison, unidirectional promoters (UDPs) were selected from unidirectional genes with expression levels similar to those of the bidirectional gene pairs for parallel analyses with BDPs. To identify DHSs located with BDPs, we first performed DHSs peak calling used F-seq software described by Boyle *et al.*^66^. We then used Perl script to analyze the relative position of DH peaks within each type of BDP. According to the profile of DHSs within BDPs, we grouped all rice BDPs into four categories: one mid-DHS, which indicates only one DH peak located near in the middle of BDPs; bi-DHSs, which indicates two separate or partially overlapping DHS peaks located within BDPs; one amesial DHS, which indicates only one DH peak asymmetrically located within BDPs; and no DHS, which indicates no DH peak identified within BDPs.

### Isolation of protoplasts from rice leave

We isolated the protoplasts following a published protocol with minor modifications^67^. Specifically, germinated rice seeds (*Oryza sativa L.*) cultivar Nipponbare were sown in soil and grown in a growth chamber with a photoperiod of 13 h of light at 26 °C and 11 h of darkness at 22 °C for 7–10 days. Green stem and sheath tissues from 80–100 rice seedlings were cut into approximately 0.5-mm strips using sharp razors. The cut strips were immediately transferred into 50-ml corning tube containing 10 ml of enzyme solution (1.5% Cellulase “Onozuka” R-10 (Yakult Pharmaceuticals, Tokyo); 0.75% Macerozyme^®^R-10 (Yakult Pharmaceuticals, Tokyo), 0.6 M mannitol; 10 mM MES, pH 5.7; 10 mM CaCl_2_; and 0.1%BSA) and underwent a vacuum treatment. After 30 min, the tube was carefully removed and placed on shaker at 50 rpm for 5–6 h in the dark for enzyme digestion. After enzyme digestion, 1 volume of W5 solution (154 mM NaCl, 125 mM CaCl_2_, 5 mM KCl and 2 mM MES, pH 5.7) was added, followed by shaking for an additional 10 min. Protoplasts were released by filtering through 35 μm nylon mesh into 50 ml round-bottom tubes. The pellets containing protoplasts were collected by centrifugation at 150 g for 2 min with a swing bucket. After washing once with W5 solution, the pellets were re-suspended using MMG solution (0.6 M mannitol, 15 mM MgCl_2_ and 4 mM MES, pH 5.7) and placed on ice for 30 min. After centrifuging at 150 g for 2 min, the pellets were re-suspended at a concentration of 2 × 10^6^ cells per milliliter using MMG solution, and the cells were counted using a hematocytometer. Unless otherwise stated, all of the above isolation processes were performed at room temperature.

### Plasmid vector preparation

In this study, all of the modified recombinant plasmids were derived from the pJIT163-hGFP vector (Supplementary Fig. S4), which contains a 35S promoter flanked by unique KpnI and HindIII restriction sites. The putative BDPs containing one DHS were amplified from rice genomic DNA using DNA oligos containing KpnI (5′ GGTAC^C 3′) and HindIII (5′ A^AGCTT 3′) restriction sites at either the 5′ or 3′ ends (Supplementary Table S5). The amplified DNA fragment was recovered from a 1.5% agarose gel. The purified DNA candidate and purified vector DNA were sequentially trimmed using KpnI (**Cat#:**1068A, Takara) and HindIII (**Cat#:**1060A, Takara), respectively. The double enzyme-cleaved DNA fragment and vector were put together for ligation using ligase (Cat#: C112-01, Vazyme) at 37 °C for 30 min. The ligated products were separated and recovered from a 1.5% agarose gel. Purified ligated vectors containing either the forward or reverse insertion of BDPs in the replacement of the original 35S promoter were used for downstream protoplast transfection.

### Protoplast transfection

We conducted PEG-mediated transfection as previously described with minor modifications^68^. Generally, 10 μg of each recombinant plasmid DNA was mixed with 100 μL protoplasts in a 2 ml round bottom tube, and 110 μl of freshly prepared PEG solution [40% (W/V) PEG 4000, 0.4 M mannitol and 0.1 M CaCl_2_] was added. After gentle mixing, the mixture was incubated at room temperature for 20 min in the dark, and 800 μl of W5 solution was slowly added. The resulting solution was gently inverted several times to mix well, immediately followed by centrifugation at 150 g for 2 min. The protoplasts pellets were gently re-suspended in 500 μl of W5 solution. Finally, transfected protoplasts were cultured in the dark at room temperature for 16–20 h. GFP signals were observed and photographed under fluorescent microscopy.

### Data analysis

All of the analyzed datasets are summarized in the Supplementary Table S6.

#### DNase-seq

Published DNase-seq datasets from seedlings were downloaded from NCBI (GSM655033)^36^. A DNaseI hypersensitive site (DHS) dataset from seedling tissue was computationally analyzed using a previously described pipeline^66^. Normalized DNase-seq reads were plotted across all of the BDPs identified above for DHS peak calling. The existence of DHSs was used to indicate the presence of potential individual promoter within BDPs.

#### RNA-seq

We downloaded publicly available RNA-seq datasets generated from seedlings (GSM655033)^36^. The expression value (FPKM) of bidirectional gene pairs was calculated using previously described approaches^36^.

#### ChIP-seq

We generated the following ChIP-seq datasets, H3K4ac (Millipore, 07-539), H3K9ac (Millipore, 07-352), H3K27ac (Abcam, ab4729), H3K27me3 (Millipore, 07-449), H3K9me1 (Millipore, 07-395) and H3K9me3 (Millipore, 07-442) from seedlings using a previously described method^36^. We downloaded four previously characterized ChIP-seq datasets from seedlings (H3K4me3, GSM489075; H3K4me2, GSM658110; H3K36me3, GSM658111 and H4K12ac; GSM658112). All of the ChIP-seq datasets were analyzed using a previously described pipeline. Normalized ChIP-seq reads were plotted across all of the bidirectional gene pairs and randomly selected unidirectional genes as controls for profiling the chromatin features of histone marks associated with bidirectional gene pairs.

#### MNase-seq

We download the MNase-seq datasets from seedlings (NCBI Sequence Read Archive (SRA), SRP045236) and analyzed the MNase-seq data using a previously described pipeline^69^. Normalized MNase-seq reads were plotted across all of the bidirectional gene pairs for profiling nucleosome positioning associated with bidirectional gene pairs.

### Coexpression analysis

Eleven expression datasets were derived from the Rice Genome Annotation Project (http://rice.plantbiology.msu.edu/expression.shtml). The raw data were extracted from the NCBI Sequence Read Archive (SRA) (Supplementary Table S2). Sequencing reads were mapped to version 7 pseudo-molecules using TopHat^70^. The expression abundances for RNA-seq libraries were calculated with Cufflinks^71^. The presence/absence of expression values were assigned for digital gene expression (DGE) libraries. For Pearson correlations, the FPKM values of bidirectional gene pairs were used for matrix analysis. Genes with FPKM = 0 across all libraries were not included for analysis. The PCCs (Pearson correlation coefficients) were calculated for each pair of bidirectional genes using a customized Perl script. For comparison, we randomly selected 1000 non-adjacent gene pairs to calculate the Pearson correlation coefficient.

### Significance test

To determine whether gene expression, histone marks and nucleosome occupancy differed significantly between BDPs and UDPs, we performed a two-sample Kolmogorov-Smirnov (K-S) test.

We first normalized the reads count distributed within BDP or UDP regions, including 1 kb downstream of TSS and promoter regions, to profile nucleosome positioning (MNase-seq reads) and histone marks. The region between TSSs was selected for BDPs, and 1 kb upstream of a TSS was chosen for UDPs. Briefly, after the identification of all of the uniquely mapped reads, we equally split the region 1 kb downstream of the TSS of BDPs and promoter or 1 kb downstream of the TSS of UDPs into 20 sliding windows with 50 bp per window. We then calculated the number of reads within a specific sliding window divided by the length of the sliding window (bp) and the number of reads within the mapped genome (million). The cumulative sum of BDPs or UDPs per sliding window was divided into the number of BDPs or UDPs that we analyzed. The midpoint of each mapped reads was used to define its position in the rice genome.

For the significance test of the difference in histone markers and nucleosome occupancy between BDPs and UDPs, we calculated the normalized reads count associated with each bidirectional gene pair and selected 1000 UDPs as controls, which are distributed either across the whole gene body or within the highest peak ranging from 100 bp to 150 bp downstream of TSS. R was used for all of the two-sample Kolmogorov-Smirnov (K-S) tests within groups, and “two.sided” was selected as the alternative hypothesis. The output of a two-tailed *p*-value less than 0.05 was considered as a significant difference between two samples.

### Data Submission

The ChIP-seq datasets has been deposited in the NCBI’s Gene Expression Omnibus (GEO) (http://www.ncbi.nlm.nih.gov/geo/) under the accession no. **GSE79033**.

## Additional Information

**How to cite this article**: Fang, Y. *et al.* Functional characterization of open chromatin in bidirectional promoters of rice. *Sci. Rep.* **6**, 32088; doi: 10.1038/srep32088 (2016).

## Supplementary Material

Supplementary Information

## Figures and Tables

**Table 1:** Distribution of DHSs within BDPs.

BDPS	one mid-DHS	one amesial DHS	bi-DHSs	no DHS	Total
BDPs-I	227 (78.27%)	22 (7.59%)	10 (3.45%)	31(10.69%)	290
BDPs-II	74 (25.17%)	92 (31.29%)	36 (12.24%)	92(31.29%)	294
BDPs-III	27 (4.31%)	237 (37.80%)	158 (25.20%)	205(32.70%)	627

## References

[b1] Wright K. L. *et al.* Coordinate regulation of the human TAP1 and LMP2 genes from a shared bidirectional promoter. J Exp Med 181, 1459–1471 (1995). 7699330 10.1084/jem.181.4.1459 PMC2191963

[b2] Guarguaglini G. *et al.* Expression of the murine RanBP1 and Htf9-c genes is regulated from a shared bidirectional promoter during cell cycle progression. Biochem J 325 (Pt 1), 277–286 (1997). 9224656 10.1042/bj3250277 PMC1218555

[b3] Momota R. *et al.* Two genes, COL4A3 and COL4A4 coding for the human alpha3(IV) and alpha4(IV) collagen chains are arranged head-to-head on chromosome 2q36. FEBS Lett 424, 11–16 (1998). 9537506 10.1016/s0014-5793(98)00128-8

[b4] Ahn J. & Gruen J. R. The genomic organization of the histone clusters on human 6p21.3. Mamm Genome 10, 768–770 (1999). 10384058 10.1007/s003359901089

[b5] Adachi N. & Lieber M. R. Bidirectional gene organization: a common architectural feature of the human genome. Cell 109, 807–809 (2002). 12110178 10.1016/s0092-8674(02)00758-4

[b6] Hansen J. J. *et al.* Genomic structure of the human mitochondrial chaperonin genes: HSP60 and HSP10 are localised head to head on chromosome 2 separated by a bidirectional promoter. Hum Genet 112, 71–77 (2003). 12483302 10.1007/s00439-002-0837-9

[b7] West A. B., Lockhart P. J., O’Farell C. & Farrer M. J. Identification of a novel gene linked to parkin via a bi-directional promoter. J Mol Biol 326, 11–19 (2003). 12547187 10.1016/s0022-2836(02)01376-1

[b8] Hogan G. J., Lee C. K. & Lieb J. D. Cell cycle-specified fluctuation of nucleosome occupancy at gene promoters. PLoS Genet 2, e158 (2006). 17002501 10.1371/journal.pgen.0020158 PMC1570381

[b9] Yang M. Q., Koehly L. M. & Elnitski L. L. Comprehensive annotation of bidirectional promoters identifies co-regulation among breast and ovarian cancer genes. PLoS Comput Biol 3, e72 (2007). 17447839 10.1371/journal.pcbi.0030072 PMC1853124

[b10] Kleinjan D. A. & Lettice L. A. Long-range gene control and genetic disease. Adv Genet 61, 339–388 (2008). 18282513 10.1016/S0065-2660(07)00013-2

[b11] Chen P. Y., Chang W. S., Lai Y. K. & Wu C. W. c-Myc regulates the coordinated transcription of brain disease-related PDCD10-SERPINI1 bidirectional gene pair. Mol Cell Neurosci 42, 23 (2009). 19442737 10.1016/j.mcn.2009.05.001

[b12] Wang G. *et al.* Identification of regulatory regions of bidirectional genes in cervical cancer. BMC Med Genomics 6 Suppl 1, S5 (2013). 23369456 10.1186/1755-8794-6-S1-S5 PMC3552671

[b13] Bondino H. G. & Valle E. M. A small intergenic region drives exclusive tissue-specific expression of the adjacent genes in Arabidopsis thaliana. BMC Mol Biol 10, 95 (2009). 19835620 10.1186/1471-2199-10-95 PMC2772851

[b14] Banerjee J., Sahoo D. K., Dey N., Houtz R. L. & Maiti I. B. An intergenic region shared by At4g35985 and At4g35987 in Arabidopsis thaliana is a tissue specific and stress inducible bidirectional promoter analyzed in transgenic arabidopsis and tobacco plants. PLoS One 8, e79622 (2013). 24260266 10.1371/journal.pone.0079622 PMC3834115

[b15] Kourmpetli S. *et al.* Bidirectional promoters in seed development and related hormone/stress responses. BMC Plant Biol 13, 187 (2013). 24261334 10.1186/1471-2229-13-187 PMC4222868

[b16] Liu S. J., Yue Q. J. & Zhang W. Structural and functional analysis of an asymmetric bidirectional promoter in Arabidopsis thaliana. J Integr Plant Biol 57, 162–170 (2015). 25370697 10.1111/jipb.12308

[b17] Khan Z. A., Abdin M. Z. & Khan J. A. Functional characterization of a strong bi-directional constitutive plant promoter isolated from cotton leaf curl Burewala virus. PLoS One 10, e0121656(2015). 25799504 10.1371/journal.pone.0121656 PMC4370823

[b18] Abdelsamad A. & Pecinka A. Pollen-specific activation of Arabidopsis retrogenes is associated with global transcriptional reprogramming. Plant Cell 26, 3299–3313 (2014). 25118244 10.1105/tpc.114.126011 PMC4371830

[b19] Xu Z. *et al.* Bidirectional promoters generate pervasive transcription in yeast. Nature 457, 1033–1037 (2009). 19169243 10.1038/nature07728 PMC2766638

[b20] Neil H. *et al.* Widespread bidirectional promoters are the major source of cryptic transcripts in yeast. Nature 457, 1038–1042 (2009). 19169244 10.1038/nature07747

[b21] Yang L. & Yu J. A comparative analysis of divergently-paired genes (DPGs) among Drosophila and vertebrate genomes. BMC Evol Biol 9, 55 (2009). 19284596 10.1186/1471-2148-9-55 PMC2670823

[b22] Trinklein N. D. *et al.* An abundance of bidirectional promoters in the human genome. Genome Res 14, 62–66 (2004). 14707170 10.1101/gr.1982804 PMC314279

[b23] Dhadi S. R., Krom N. & Ramakrishna W. Genome-wide comparative analysis of putative bidirectional promoters from rice, Arabidopsis and Populus. Gene 429, 65–73 (2009). 18973799 10.1016/j.gene.2008.09.034

[b24] Liu X. *et al.* Identification and functional characterization of bidirectional gene pairs and their intergenic regions in maize. BMC Genomics 15, 338 (2014). 24886269 10.1186/1471-2164-15-338 PMC4035068

[b25] Bornelov S., Komorowski J. & Wadelius C. Different distribution of histone modifications in genes with unidirectional and bidirectional transcription and a role of CTCF and cohesin in directing transcription. BMC Genomics 16, 300 (2015). 25881024 10.1186/s12864-015-1485-5 PMC4446127

[b26] Lin J. M. *et al.* Transcription factor binding and modified histones in human bidirectional promoters. Genome Res 17, 818–827 (2007). 17568000 10.1101/gr.5623407 PMC1891341

[b27] Wakano C., Byun J. S., Di L. J. & Gardner K. The dual lives of bidirectional promoters. Biochim Biophys Acta 1819, 688–693 (2012). 22366276 10.1016/j.bbagrm.2012.02.006 PMC3371153

[b28] Krom N. & Ramakrishna W. Comparative analysis of divergent and convergent gene pairs and their expression patterns in rice, Arabidopsis, and populus. Plant Physiol 147, 1763–1773 (2008). 18515639 10.1104/pp.108.122416 PMC2492640

[b29] Yang X. H., W C., Xia X. Y. & Gang S. H. Genome-wide analysis of intergenic regions in Arabidopsis thaliana suggests the existence of bidirectional promoters and genetic insulators. Current Topics in Plant Biology 12, 15–33 (2011).

[b30] Wang Q. *et al.* Searching for bidirectional promoters in Arabidopsis thaliana. BMC Bioinformatics 10 Suppl 1, S29 (2009). 19208129 10.1186/1471-2105-10-S1-S29 PMC2648788

[b31] Dhadi S. R., Deshpande A., Driscoll K. & Ramakrishna W. Major cis-regulatory elements for rice bidirectional promoter activity reside in the 5′-untranslated regions. Gene 526, 400–410 (2013). 23756196 10.1016/j.gene.2013.05.060

[b32] Chen W. H., de Meaux J. & Lercher M. J. Co-expression of neighbouring genes in Arabidopsis: separating chromatin effects from direct interactions. BMC Genomics 11, 178 (2010). 20233415 10.1186/1471-2164-11-178 PMC2851598

[b33] Boyle A. P. *et al.* High-resolution mapping and characterization of open chromatin across the genome. Cell 132, 311–322 (2008). 18243105 10.1016/j.cell.2007.12.014 PMC2669738

[b34] Kharchenko P. V. *et al.* Comprehensive analysis of the chromatin landscape in Drosophila melanogaster. Nature 471, 480–485 (2011). 21179089 10.1038/nature09725 PMC3109908

[b35] Thurman R. E. *et al.* The accessible chromatin landscape of the human genome. Nature 489, 75–82 (2012). 22955617 10.1038/nature11232 PMC3721348

[b36] Zhang W. *et al.* High-resolution mapping of open chromatin in the rice genome. Genome Res 22, 151–162 (2012). 22110044 10.1101/gr.131342.111 PMC3246202

[b37] Bell O., Tiwari V. K., Thoma N. H. & Schubeler D. Determinants and dynamics of genome accessibility. Nat Rev Genet 12, 554–564 (2011). 21747402 10.1038/nrg3017

[b38] Batada N. N., Urrutia A. O. & Hurst L. D. Chromatin remodelling is a major source of coexpression of linked genes in yeast. Trends Genet 23, 480–484 (2007). 17822800 10.1016/j.tig.2007.08.003

[b39] He H. H. *et al.* Refined DNase-seq protocol and data analysis reveals intrinsic bias in transcription factor footprint identification. Nat Methods 11, 73–78 (2014). 24317252 10.1038/nmeth.2762 PMC4018771

[b40] Sproul D., Gilbert N. & Bickmore W. A. The role of chromatin structure in regulating the expression of clustered genes. Nat Rev Genet 6, 775–781 (2005). 16160692 10.1038/nrg1688

[b41] Yuan G. C. *et al.* Genome-scale identification of nucleosome positions in S. cerevisiae. Science 309, 626–630 (2005). 15961632 10.1126/science.1112178

[b42] Schones D. E. *et al.* Dynamic regulation of nucleosome positioning in the human genome. Cell 132, 887–898 (2008). 18329373 10.1016/j.cell.2008.02.022 PMC10894452

[b43] Jiang C. & Pugh B. F. Nucleosome positioning and gene regulation: advances through genomics. Nat Rev Genet 10, 161–172 (2009). 19204718 10.1038/nrg2522 PMC4860946

[b44] Wan J., Lin J., Zack D. J. & Qian J. Relating periodicity of nucleosome organization and gene regulation. Bioinformatics 25, 1782–1788 (2009). 19447785 10.1093/bioinformatics/btp323 PMC2705233

[b45] Bai L. & Morozov A. V. Gene regulation by nucleosome positioning. Trends Genet 26, 476–483 (2010). 20832136 10.1016/j.tig.2010.08.003

[b46] Tsankov A. M., Thompson D. A., Socha A., Regev A. & Rando O. J. The role of nucleosome positioning in the evolution of gene regulation. PLoS Biol 8, e1000414 (2010). 20625544 10.1371/journal.pbio.1000414 PMC2897762

[b47] Rhie S. K. *et al.* Nucleosome positioning and histone modifications define relationships between regulatory elements and nearby gene expression in breast epithelial cells. BMC Genomics 15, 331 (2014). 24885402 10.1186/1471-2164-15-331 PMC4035062

[b48] Liu M. J. *et al.* Determinants of nucleosome positioning and their influence on plant gene expression. Genome Res 25, 1182–11195 (2015). 26063739 10.1101/gr.188680.114 PMC4510002

[b49] Cui P. *et al.* A quantitative analysis of the impact on chromatin accessibility by histone modifications and binding of transcription factors in DNase I hypersensitive sites. Biomed Res Int 2013, 914971 (2013). 24236298 10.1155/2013/914971 PMC3819824

[b50] Rollini P. & Fournier R. E. Differential regulation of gene activity and chromatin structure within the human serpin gene cluster at 14q32.1 in macrophage microcell hybrids. Nucleic Acids Res 28, 1767–1777 (2000). 10734196 10.1093/nar/28.8.1767 PMC102814

[b51] Saunders A., Core L. J. & Lis J. T. Breaking barriers to transcription elongation. Nat Rev Mol Cell Biol 7, 557–567 (2006). 16936696 10.1038/nrm1981

[b52] Kuras L. & Struhl K. Binding of TBP to promoters *in vivo* is stimulated by activators and requires Pol II holoenzyme. Nature 399, 609–613 (1999). 10376605 10.1038/21239

[b53] Turner B. M. Cellular memory and the histone code. Cell 111, 285–291 (2002). 12419240 10.1016/s0092-8674(02)01080-2

[b54] Bernstein B. E. *et al.* Genomic maps and comparative analysis of histone modifications in human and mouse. Cell 120, 169–181 (2005). 15680324 10.1016/j.cell.2005.01.001

[b55] Pokholok D. K. *et al.* Genome-wide map of nucleosome acetylation and methylation in yeast. Cell 122, 517–527 (2005). 16122420 10.1016/j.cell.2005.06.026

[b56] Schneider R. *et al.* Histone H3 lysine 4 methylation patterns in higher eukaryotic genes. Nat Cell Biol 6, 73–77 (2004). 14661024 10.1038/ncb1076

[b57] Bintu L. *et al.* Nucleosomal elements that control the topography of the barrier to transcription. Cell 151, 738–749 (2012). 23141536 10.1016/j.cell.2012.10.009 PMC3508686

[b58] Zentner G. E. & Henikoff S. Regulation of nucleosome dynamics by histone modifications. Nat Struct Mol Biol 20, 259–266 (2013). 23463310 10.1038/nsmb.2470

[b59] Jonkers I. & Lis J. T. Getting up to speed with transcription elongation by RNA polymerase II. Nat Rev Mol Cell Biol 16, 167–177 (2015). 25693130 10.1038/nrm3953 PMC4782187

[b60] Wu Y., Zhang W. & Jiang J. Genome-wide nucleosome positioning is orchestrated by genomic regions associated with DNase I hypersensitivity in rice. PLoS Genet 10, e1004378 (2014). 24852592 10.1371/journal.pgen.1004378 PMC4031139

[b61] Radman-Livaja M. & Rando O. J. Nucleosome positioning: how is it established, and why does it matter? Dev Biol 339, 258–266 (2010). 19527704 10.1016/j.ydbio.2009.06.012 PMC2830277

[b62] Gaffney D. J. *et al.* Controls of nucleosome positioning in the human genome. PLoS Genet 8, e1003036 (2012). 23166509 10.1371/journal.pgen.1003036 PMC3499251

[b63] Zhang Y. *et al.* Intrinsic histone-DNA interactions are not the major determinant of nucleosome positions *in vivo*. Nat Struct Mol Biol 16, 847–852 (2009). 19620965 10.1038/nsmb.1636 PMC2823114

[b64] Rhee H. S. & Pugh B. F. Genome-wide structure and organization of eukaryotic pre-initiation complexes. Nature 483, 295–301 (2012). 22258509 10.1038/nature10799 PMC3306527

[b65] Vaillant C. *et al.* A novel strategy of transcription regulation by intragenic nucleosome ordering. Genome Res 20, 59–67 (2010). 19858362 10.1101/gr.096644.109 PMC2798831

[b66] Boyle A. P., Guinney J., Crawford G. E. & Furey T. S. F-Seq: a feature density estimator for high-throughput sequence tags. Bioinformatics 24, 2537–2538 (2008). 18784119 10.1093/bioinformatics/btn480 PMC2732284

[b67] Zhang Y. *et al.* A highly efficient rice green tissue protoplast system for transient gene expression and studying light/chloroplast-related processes. Plant Methods 7, 30 (2011). 21961694 10.1186/1746-4811-7-30 PMC3203094

[b68] Yoo S. D., Cho Y. H. & Sheen J. Arabidopsis mesophyll protoplasts: a versatile cell system for transient gene expression analysis. Nat Protoc 2, 1565–1572 (2007). 17585298 10.1038/nprot.2007.199

[b69] Zhang T., Zhang W. & Jiang J. Genome-Wide Nucleosome Occupancy and Positioning and Their Impact on Gene Expression and Evolution in Plants. Plant Physiol 168, 1406–14416 (2015). 26143253 10.1104/pp.15.00125 PMC4528733

[b70] Trapnell C., Pachter L. & Salzberg S. L. TopHat: discovering splice junctions with RNA-Seq. Bioinformatics 25, 1105–1111 (2009). 19289445 10.1093/bioinformatics/btp120 PMC2672628

[b71] Trapnell C. *et al.* Transcript assembly and quantification by RNA-Seq reveals unannotated transcripts and isoform switching during cell differentiation. Nat Biotechnol 28, 511–515 (2010). 20436464 10.1038/nbt.1621 PMC3146043

