# Supplementary material for: Functional characterization of open chromatin in bidirectional promoters of rice
Source: Sci Rep. 2016 Aug 25;6:32088. doi: 10.1038/srep32088 (PMC4997330; doi:10.1038/srep32088)
Supplement: Supplementary Information [file srep32088-s1.pdf]

Supplementary information:

Functional characterization of open chromatin in bidirectional promoters of rice

Yuan Fang, Ximeng Wang, Lei Wang,Xiucai Pan, Jin Xiao, Xiu-e Wang, Yufeng Wu, Wenli Zhang*

State Key Laboratory for Crop Genetics and Germplasm Enhancement, JiangSu Collaborative Innovation Center for Modern Crop Production (JCIC-MCP), Nanjing Agriculture University, Nanjing, Jiangsu 210095, China

* To whom correspondence should be addressed. E-mail: wzhang25@njau.edu.cn

Running Title: open chromatin associated with rice bidirectional promoters

*Authors for correspondence

Wenli Zhang
E-mail: wzhang25@njau.edu.cn
Tel: +86-25-84399893
Fax: +86-25-84395526

Supplementary Figure legend:

Supplementary Figure S1. Profile of DHS distribution within BDPs

(**a**) **One mid-DHS:** DHS peak located in the middle of a BDP; (**b**) **One amesial DHS:** DHS peak located closer to one gene than to the counterpart; (**c**) **bi-DHSs**: two DHS peaks located within a BDP; (**d**) **no DHS:** no DHS peak in a BDP.

Supplementary Figure S2. Representative images of protoplast transient transfection from a BDP containing one DHS

A BDP from a bidirectional gene pair (LOC_Os02g33070 - LOC_Os02g33080) was amplified using DNA oligos containing either Kpn I or Hind III restriction sites at the 5' and 3' ends. The purified DNA fragment and vectors were sequentially trimmed using the restriction enzymes KpnI and HindIII and ligated with each other. Either the forward (F) (**a**) or reverse(R) (**b**) insertion of a BDP-related DNA fragment into the vector for the replacement of 35S promoter was chosen for the protoplast transient transfection. GFP signals were observed and recorded under fluorescent microscopy. The scale bar equals 100 µm.

Supplementary Figure S3. Coexpression analysis of gene pairs associated with BDPs with size separated by 200 bp in intergenic length

The Pearson correlation coefficient was calculated from all of the gene pairs corresponding to BDPs separated every 200 bp using the absolute expression value. Statistical analysis was provided by a two-sample K-S test, where **$p<1e-06$.

Supplementary Figure S4. Diagram of modified plasmid vectors used for transient transfections

Purified PCR-amplified BDPs and vector DNA were sequentially digested using Kpn I and Hind III followed by ligation, resulting in the replacement of the 35S promoter with amplified BDPs. The resulting ligated vector containing either the forward or reverse insertion of BDPs was used for protoplast transient transfection.

Supplementary Figure S5. Effect of DHSs on the positioning of histone marks and gene expression

The effect of the physical position of DHSs relative to the TSS of genes on the positioning of parts of marks: (**a**) H3K27me3, (**b**) H3K9me3, **c:** H3K9me1, (**d**) H3K4me2, **e:** Heatmap of the expression level of the genes indicated using FPKM proximal and distal to DHS in one amesial DHS. The X-axes in (**a-d**) show the relative distance of BDPs (bp); the Y-axes in (**a-d**) show the normalized ChIP-seq reads counts (read number per base pair in a genomic region per million reads) within ±1 kb of the TSS. We used different y-axis scales to better visualize the profile of H3K27me3 (**S5a**) and H3K4me2(**S5d**) distributed among bidirectional promoters containing different DHS.

Supplementary Figure S6. The relationship between the enrichment of histone marks and the levels of gene expression

Non-TE Genes were divided into six bins based on their expression levels (FPKM), including no expression (FPKM = 0), and from the bottom 20% to the top 100%, as indicated. ChIP-seq reads from each mark were plotted across genes with different expression levels from 1000 bp upstream and downstream of TSS. (**a**)H3K4ac; (**b**) H3K9ac; (**c**) H3K27ac; (**d**) H3K9me1; (**e**) H3K9me3 and (**f**) H3K27me3. Positive correlation and anti-correlation between marks enrichment and gene expression levels were observed in active (H3K4ac, H3K9ac and H3K27ac)

and repressive (H3K9me1, H3K9me3 and H3K27me3) marks, respectively. We used different y-axis scales to better visualize the profile of H3K27ac (**S6 c**) and H3K27me3 (**S6 f**) distributed among different expression levels of genes.

Table 1. Distribution of DHSs within BDPs

Supplementary Table S1. BDPs associated with genomic loci of bidirectional gene pairs selected for protoplast transfection

Supplementary Table S2. Information of downloaded expression datasets

Supplementary Table S3. Effect of DHS distribution on the coexpression of bidirectional gene pairs separated every 100 bp

Supplementary Table S4. Effect of DHS distribution on the coexpression of bidirectional gene pairs in BDP I/II/III

Supplementary Table S5. DNA oligos that were used for the PCR amplification of BDP candidates (red letters represent restriction enzyme sites)

Supplementary Table S6. Sequencing data information

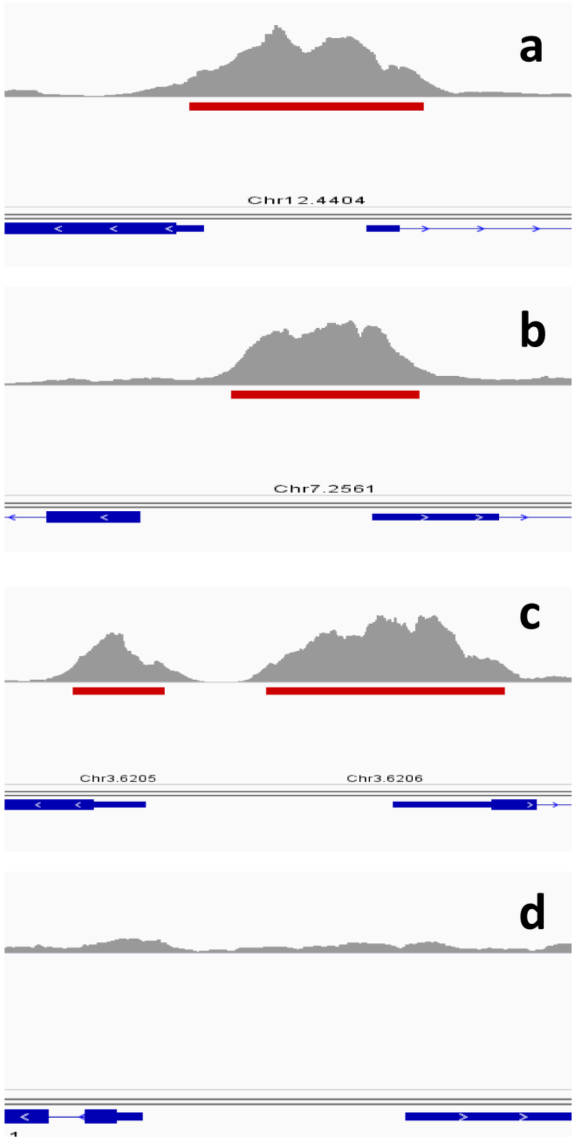
a
Chr12.4404
b
Chr7.2561
c
Chr3.6205
Chr3.6206
d

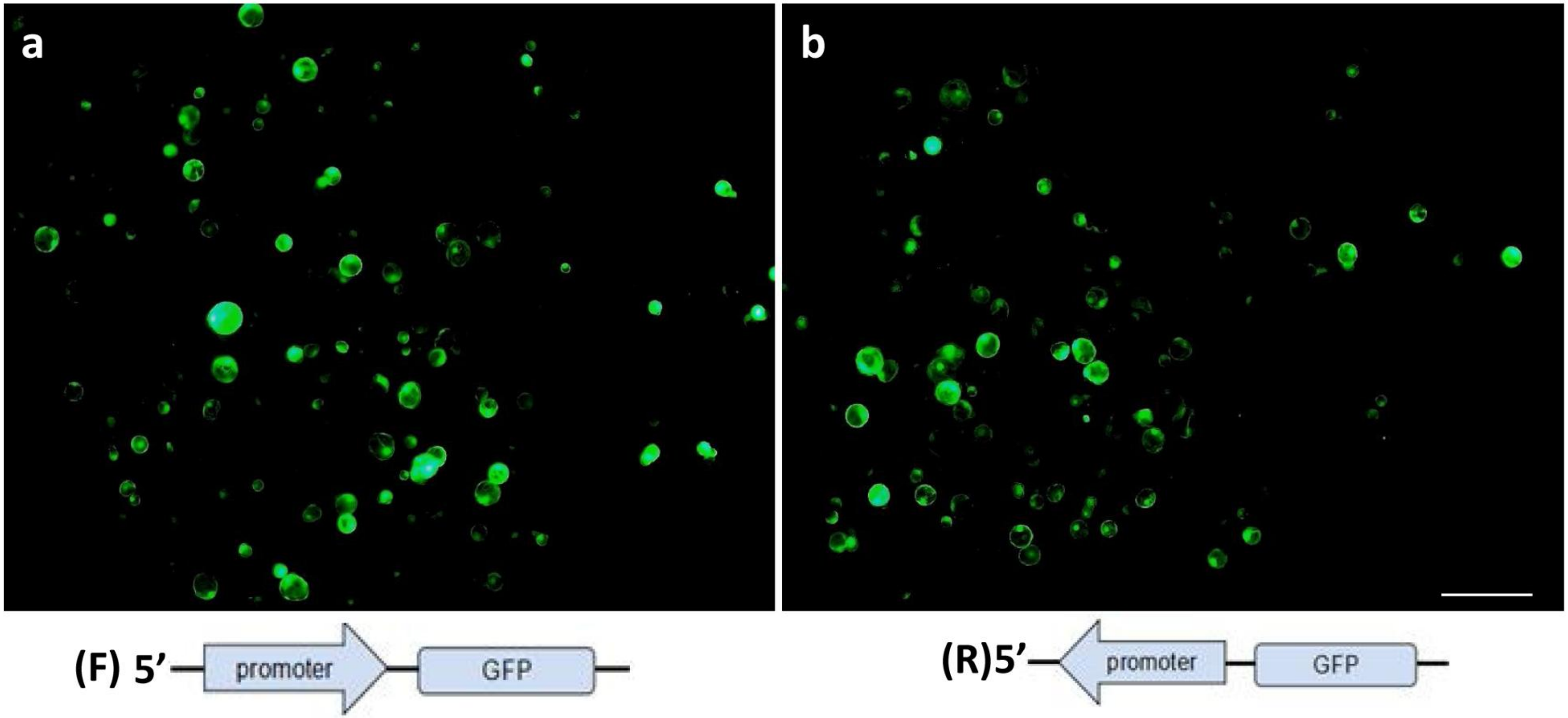
a
b
(F) 5'
promoter
GFP
(R)5'
promoter
GFP

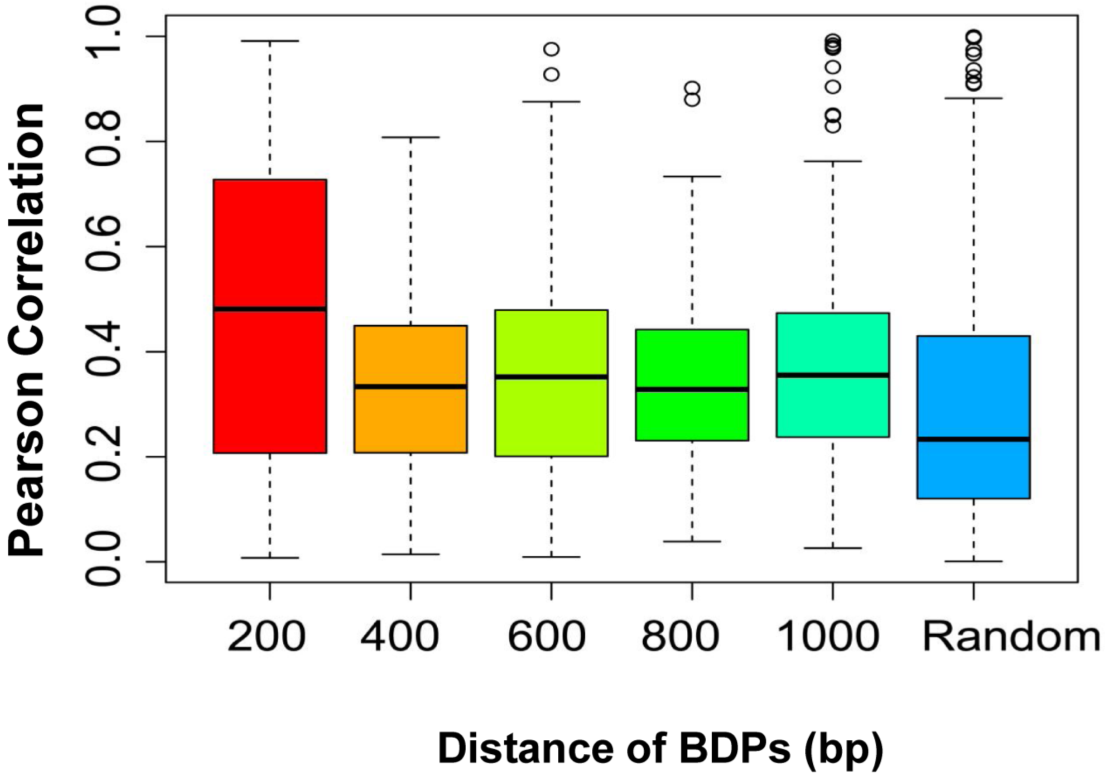

Note: significant test shows that change of coexpression between 200bp and 400-1000bp is significant (*p* value<0.01); change of coexpression between BDPs with every 200bp and random is significant (*p* value is 4.219e-15, 7.858e-07, 6.787e-08, 1.352e-07 and 2.256e-09, respectively.

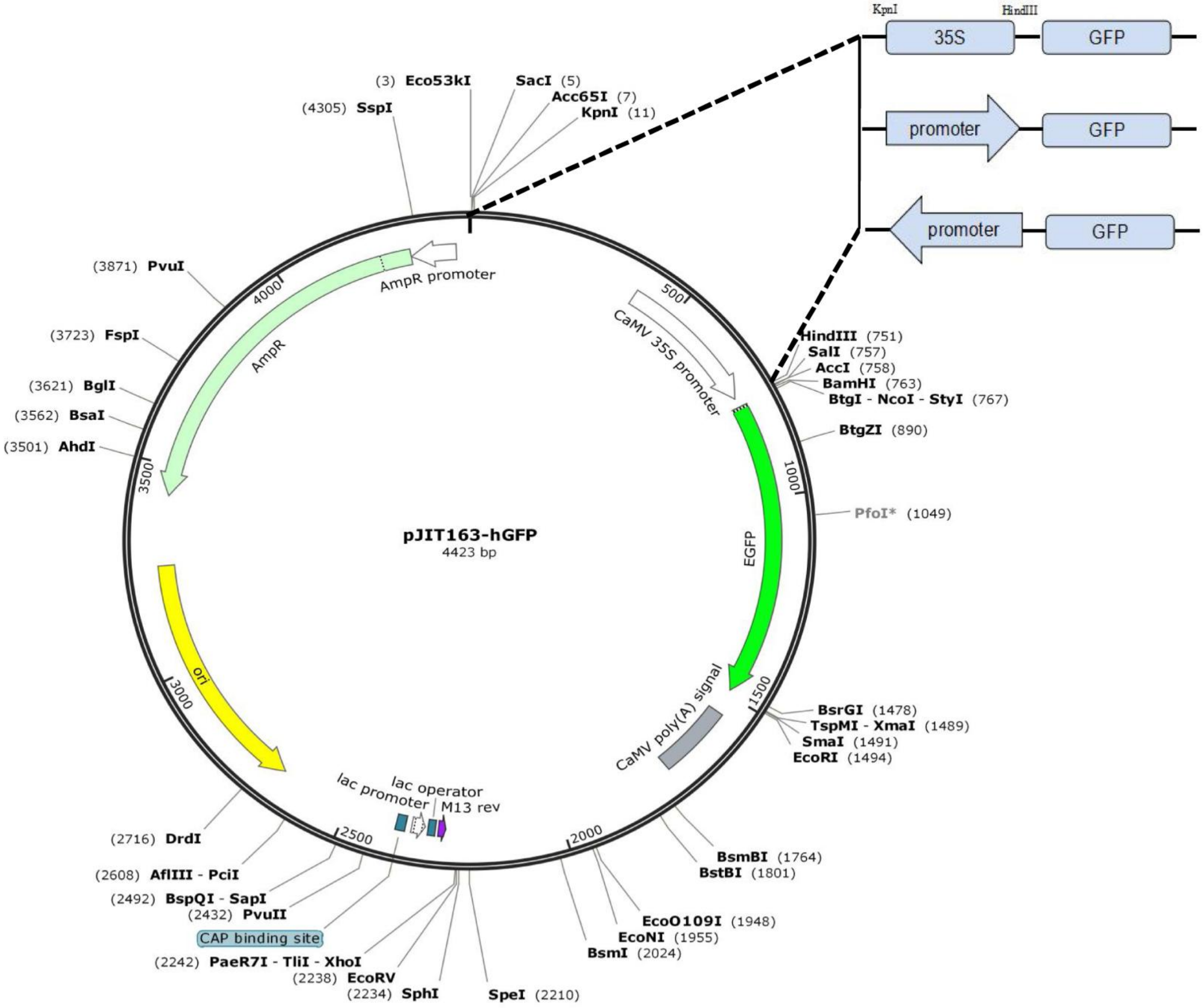

KpnI
HindIII
35S
GFP
promoter
GFP
promoter
GFP
(3) Eco53kI
SacI (5)
Acc65I (7)
KpnI (11)
(4305) SspI
(3871) PvuI
(3723) FspI
(3621) BglI
(3562) BsaI
(3501) AhdI
AmpR promoter
AmpR
CaMV 35S promoter
EGFP
HindIII (751)
SalI (757)
AccI (758)
BamHI (763)
BtgI - NcoI - StyI (767)
BtgZI (890)
PfoI* (1049)
pJIT163-hGFP
4423 bp
ori
CaMV poly(A) signal
BsrGI (1478)
TspMI - XmaI (1489)
SmaI (1491)
EcoRI (1494)
lac promoter
lac operator
M13 rev
(2716) DrdI
(2608) AflIII - PciI
(2492) BspQI - SapI
(2432) PvuII
CAP binding site
(2242) PaeR7I - TliI - XhoI
(2238) EcoRV
(2234) SphI
SpeI (2210)
BsmBI (1764)
BstBI (1801)
EcoO109I (1948)
EcoNI (1955)
BsmI (2024)
500
1000
1500
2000
2500
3000
3500
4000

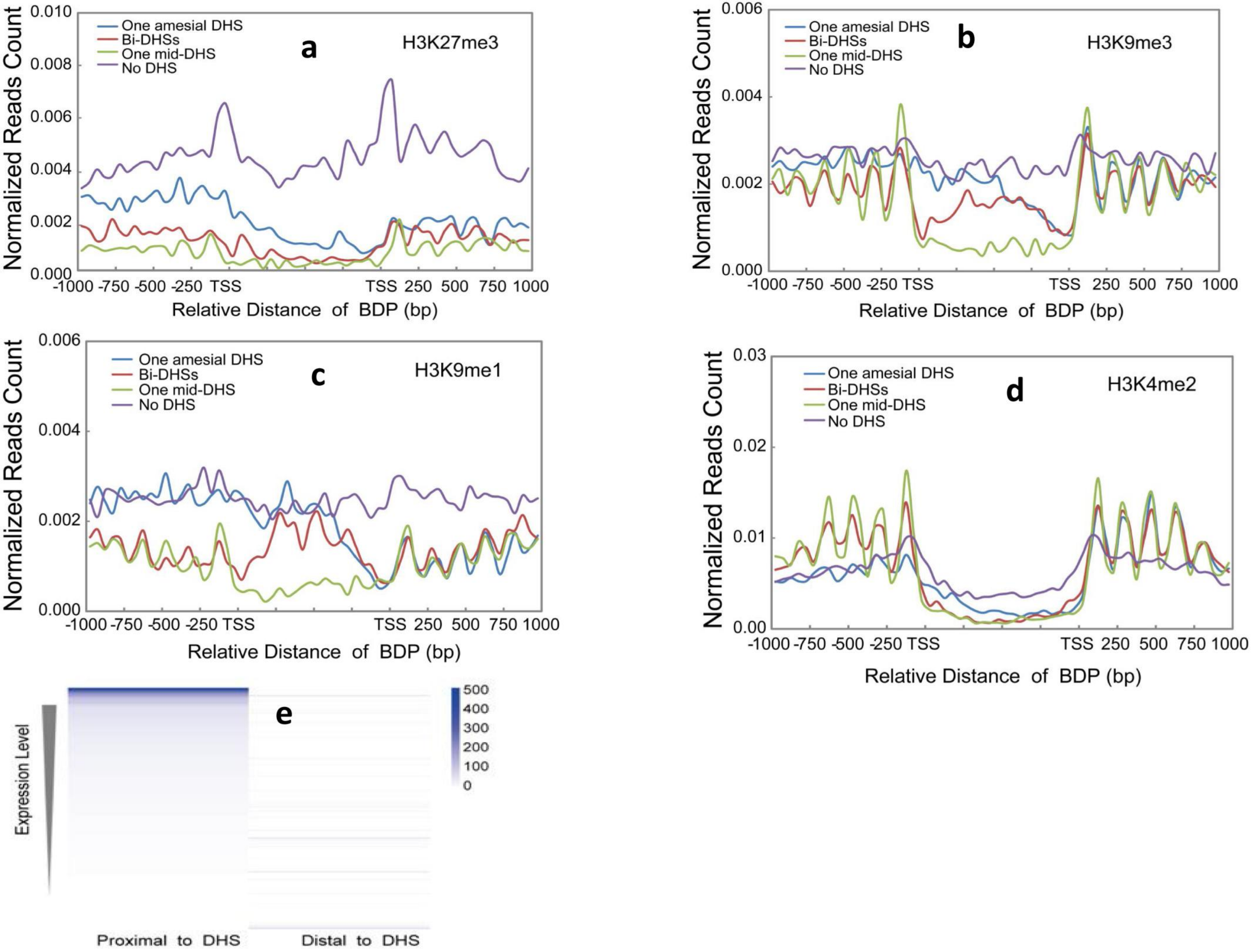
a
H3K27me3
b
H3K9me3
c
H3K9me1
d
H3K4me2
e
One amesial DHS
Bi-DHSs
One mid-DHS
No DHS
Normalized Reads Count
Relative Distance of BDP (bp)
-1000 -750 -500 -250 TSS
TSS 250 500 750 1000
0.000
0.002
0.004
0.006
0.008
0.010
0.00
0.01
0.02
0.03
Expression Level
Proximal to DHS
Distal to DHS
500
400
300
200
100
0

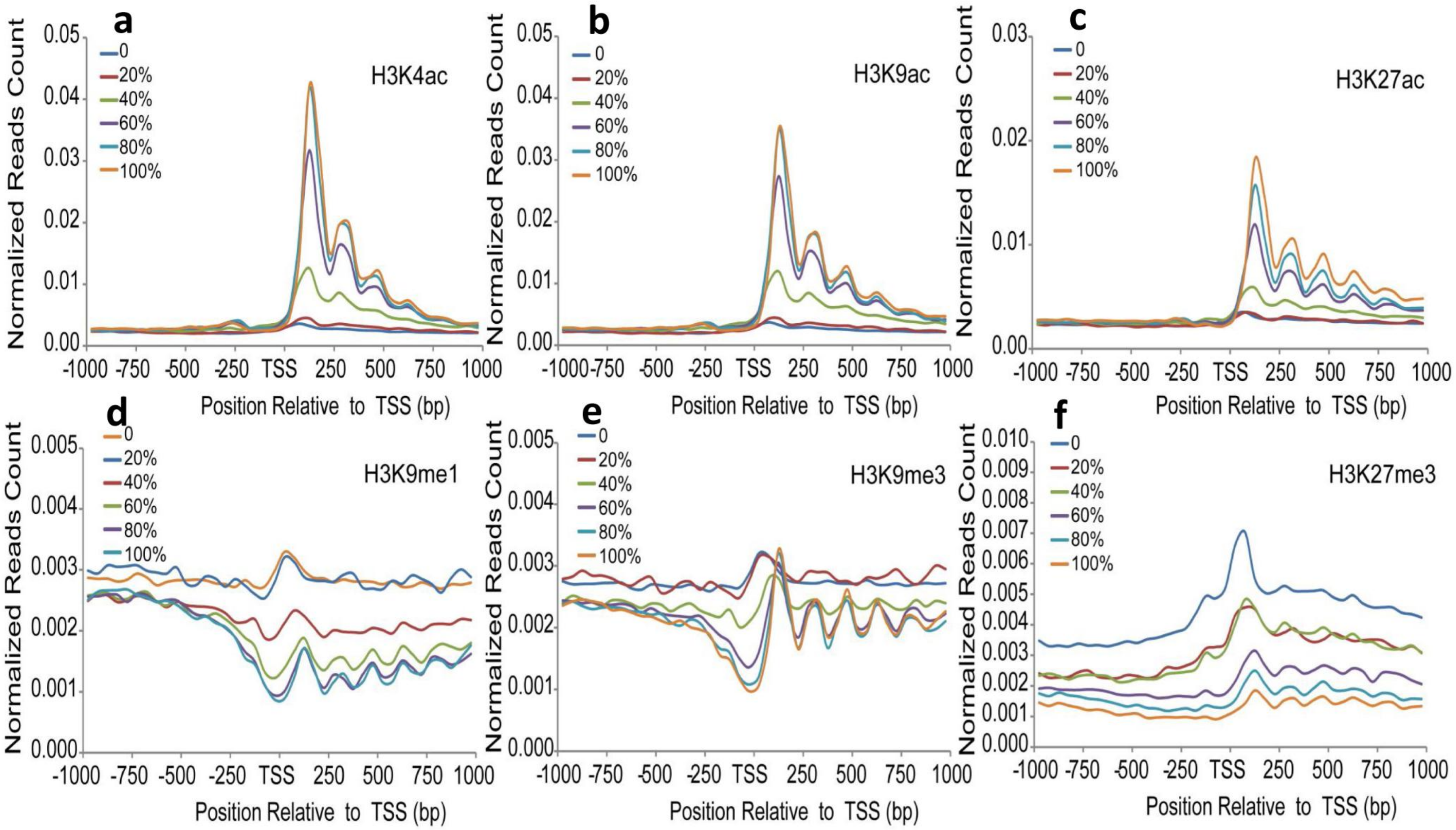
a
H3K4ac
b
H3K9ac
c
H3K27ac
d
H3K9me1
e
H3K9me3
f
H3K27me3
0
20%
40%
60%
80%
100%
Normalized Reads Count
Position Relative to TSS (bp)
-1000
-750
-500
-250
TSS
250
500
750
1000
